# An Analysis of the Nutritional Adequacy of Mass-Marketed Vegan Recipes

**DOI:** 10.7759/cureus.37131

**Published:** 2023-04-04

**Authors:** Mary Graham, Cynthia Clark, Andie Scherer, Mark Ratner, Carl Keen

**Affiliations:** 1 Nutrition, Theralogix LLC, Rockville, USA; 2 Nutrition, University of California Davis, Davis, USA

**Keywords:** nutrient deficiency, mineral, vitamin, vegetarian, vegan, plant-based diet

## Abstract

The popularity of plant-based and vegan diets has grown in recent years. While following a vegan diet has been associated with various health benefits, the intake of certain vitamins and minerals including vitamins B12 and D, calcium, and iron, are likely to be inadequate in an exclusively plant-based diet. Low nutrient intake over time can lead to nutritional deficiencies and potentially increased risk of adverse health outcomes. In this study, we analyzed a one-week meal plan comprised of vegan recipes from Forks Over Knives (FOK), an organization that promotes a low-fat, whole-food, vegan diet to prevent or reverse chronic disease. A detailed analysis of the meal plan found that it was deficient in several nutrients. It did not meet at least 90% of the daily value (DV) for the following: biotin (56% DV), calcium (58% DV), choline (30% DV), iodine (1% DV), niacin (75%), selenium (68%), vitamin B12 (82% DV), vitamin D (5% DV), vitamin E (7% DV), and zinc (64% DV). Based on evidence from this analysis, vegans and their healthcare providers should be aware of potential nutrient deficiencies and health consequences that may result from this type of diet.

## Introduction

Plant-based diets have increased in popularity over the last several years for many reasons including environmental, ethical, and health concerns. Exclusively plant-based or vegan diets, which exclude all animal foods and any food from an animal, are slowly gaining mainstream popularity. U.S. sales of plant-based dairy and meat alternatives increased 27% from 2019 to 2020, with a total plant-based market value estimated at $7 billion [1]. The global market for dairy alternatives is expected to reach $25 billion by 2026 [2]. One survey found a 300% increase in the number of people who consider themselves vegans between 2004 and 2019, amounting to about 3% of the total population or nearly 10 million people [3]. According to the Vegetarian Resource Group 2020 Harris Poll, 3% of U.S. adults are vegan and 6% are vegetarian (including vegan) [4].

The potential health benefits of plant-based diets are well documented. They typically contain higher amounts of fruits, vegetables, whole grains, nuts, soy products, and phytochemicals. Plant-based diets are generally lower in saturated fat and cholesterol and higher in dietary fiber [5]. Plant-based diets are associated with a lower risk of death from ischemic heart disease, lower low-density lipoprotein (LDL) cholesterol and blood pressure levels, and lower rates of hypertension and type 2 diabetes than nonvegetarian diets [5]. Vegans and vegetarians also tend to have a lower body mass index and overall cancer rate than omnivores [5]. In addition, randomized controlled trials of vegan and vegetarian interventions have shown improvements in body weight and cardiometabolic risk factors [6].

Despite these many health advantages of plant-based diets, research indicates that vegan diets are generally low in certain nutrients, potentially resulting in nutrient deficiencies and related negative health effects over time, including an increased risk of bone fractures, vitamin B12 deficiency and peripheral neuropathy, and hemorrhagic stroke [6-8]. A systematic review found vegan diets low in vitamin B2, niacin (B3), vitamin B12, vitamin D, iodine, zinc, calcium, potassium, and selenium [9]. Another systematic review of 141 studies showed that vegans and vegetarians had lower intakes of eicosapentaenoic acid (EPA) and docosahexaenoic acid (DHA) when compared to meat-eaters. This review also found that intakes and status of vitamin B12, vitamin D, iron, zinc, iodine, and calcium were lower in plant-based diets. Those on vegan diets had the lowest intakes of vitamin B12, calcium, and iodine, as well as lower bone mineral density (BMD) [10].

Consumers have access to thousands of resources for vegan recipes including websites, YouTube videos, books, magazines, and more. One of these resources is Forks Over Knives (FOK), an organization known for its 2011 documentary that advocated a low-fat, whole-food, vegan diet to prevent or reverse several chronic diseases. The FOK diet avoids overly refined and processed foods, including refined sugars, bleached flours, and oils, and instead includes predominantly whole grains, legumes, tubers, vegetables, and fruits. Since 2011, FOK has released several books, offers a mobile recipe app, a magazine, a line of food products, and maintains a website with research, recipes, and tools to help people with their plant-based meal planning [11].

Here, we present the results of a nutrient analysis of a one-week, 2,000 daily calorie, vegan meal plan comprised solely of FOK recipes. We review the nutrients that were found to be deficient by the recipe analysis, describe the potential health effects associated with insufficient intake of these nutrients, and outline the potential need for selective nutritional supplementation when appropriate.

## Materials and methods

We analyzed a meal plan created from one week of FOK vegan meal and snack recipes, 35 in total, using Nutritionist Pro version 8.1.0 software. We chose FOK recipes for our analysis because they are easily accessible to consumers in various locations such as the FOK website, mobile app, and print magazine sold in grocery stores. These plant-based recipes appear to be carefully curated and likely appeal to a wide range of consumers. The recipes included were chosen randomly from the FOK magazine's 100 Best Plant-Based Recipes of 2021 issue. Each day included breakfast, lunch, snacks, dinner, and dessert. Serving sizes were determined so that the total daily calorie intake would average 2,000, as the percent Daily Value (DV) is based on a 2,000-calorie diet The recipes included in the analysis are shown in Table 1.

**Table 1:** One week of FOK vegan recipes

Day	Breakfast	Lunch	Snack	Dinner	Dessert
Day 1	Berry Banana Smoothie Bowls	"No Tuna" Salad Sandwiches	Crispy Potato Cheese Sticks	Charred Cauliflower and Red Onion Tacos	Almond Fudge Brownie Pie
Day 2	Blueberry-Poppyseed Pancakes	Middle Eastern Pita Pocket Sandwiches	Jerk-Seasoned Plantain Chips	Confetti Corn Pasta Bowl	Mini Peach Tarts
Day 3	Blueberry-Tangerine Oats	Citrus-Quinoa Salad	Mexican 11-Layer Dip	Creamy Avocado-Kale Pasta	Apple Maple Cinnamon Rolls
Day 4	Rainbow Sweet Potato Breakfast Bowls	Italian Chopped Salad with Farro	Nachos Verdes	Mexican Grain Bowls	Maple-Ginger Spiced Pear Crisp
Day 5	Steel-Cut Oats with Savory Greens	Layered Southwestern Salad	Roasted Strawberry Bruschetta	Spaghetti Marinara with Lentil Balls	Mexican Chocolate Nice Cream
Day 6	Tropical Overnight Oats	Nourishing Noodle Soup	Sweet Potato-Jalapeno Poppers	Best-Ever Beefless Stew	Strawberry Dessert Pizza
Day 7	Zesty Kale and Couscous Breakfast Bowl	Tortilla Soup	Ginger-Peach Muffins	Lentil Sloppy Joes	Perfect Vegan Carrot Cake

The Nutritionist Pro analysis included calories, protein and amino acids, carbohydrates, fat and fatty acids, vitamins, and minerals. The nutrient goal criteria for this analysis were to assess which micronutrients met 90% of the DV for adults and children over four years of age.

## Results

For macronutrients, the meal plan met 100% of the DV for calories, 140% of the DV for protein, 127% of the DV for carbohydrates, 237% of the DV for dietary fiber, 54% of the DV for total fat, and 29% of the DV for saturated fat. The nutrient analysis results for macronutrients are shown in Table 2.

**Table 2:** FOK nutrient analysis results - macronutrients Daily Value (DV) = Reference amounts (expressed in grams, milligrams, or micrograms) of nutrients to consume or not to exceed each day. Percent Daily Value (%DV) = The average percentage of the Daily Value for each nutrient.

Nutrient	Meal Plan Daily Average	Daily Value (DV)	Percent Daily Value (% DV)
Macronutrients:			
Calories	1993 kcal	2000 kcal	100%
Protein	70 g	50 g	140%
Carbohydrate	350 g	275 g	127%
Dietary fiber	66 g	28 g	237%
Total fat	42 g	78 g	54%
Saturated fat	6 g	20 g	29%

The meal plan was deficient in several micronutrients. It did not meet at least 90% of the DV for the following vitamins: biotin (56% DV), choline (30% DV), niacin (75% DV), vitamin B12 (82% DV), vitamin D (5% DV), and vitamin E (7% DV) [11]. The nutrient analysis results for vitamins are shown in Table 3.

**Table 3:** FOK nutrient analysis results - vitamins Daily Value (DV) = Reference amounts (expressed in grams, milligrams, or micrograms) of nutrients to consume or not to exceed each day. Percent Daily Value = The average percentage of the Daily Value for each nutrient. * Choline is a vitamin-like essential nutrient. +  DFE is the unit of measure for folate that takes into account absorption of folic acid versus food folate.

Nutrient	Meal Plan Daily Average	Daily Value (DV)	Percent Daily Value (% DV)
Vitamins:			
Biotin	16.762 mcg	30 mcg	56%
Choline*	162.820 mg	550 mg	30%
Folate	593.036 mcg DFE^+^	400 mcg DFE^+^	148%
Niacin	14.947 mg	20 mg	75%
Pantothenic acid	4.439 mg	5 mg	91%
Riboflavin	2.253 mg	1.3 mg	173%
Thiamin	2.311 mg	1.2 mg	193%
Vitamin A	999.605 mcg	900 mcg	111%
Vitamin B_12_	1.959 mcg	2.4 mcg	82%
Vitamin B_6_	2.685 mg	1.7 mg	158%
Vitamin C	249.963 mg	90 mg	278%
Vitamin D	1.006 mcg	20 mcg	5%
Vitamin E	1.317 mg	20 mg	7%
Vitamin K	333.362 mcg	120 mcg	278%

The meal plan did not meet at least 90% of the DV for the following minerals: calcium (58% DV), iodine (1% DV), selenium (68%), and zinc (64% DV) [11]. Although the meal plan met the DV for iron, it did not meet the adjusted Recommended Dietary Allowance (RDA) for vegetarian and vegan adults for iron [12]. The nutrient analysis results for minerals are shown in Table 4.

**Table 4:** FOK nutrient analysis results - minerals Daily Value (DV) = Reference amounts (expressed in grams, milligrams, or micrograms) of nutrients to consume or not to exceed each day. Percent Daily Value (%DV) = The average percentage of the Daily Value for each nutrient.

Nutrient	Meal Plan Daily Average	Daily Value (DV)	Percent Daily Value (% DV)
Minerals:			
Calcium	756.227 mg	1300 mg	58%
Chromium	0.068 mg	0.035 mg	194%
Iodine	1.406 mcg	150 mcg	1%
Iron	21.670 mg	18 mg	120%
Magnesium	437.119 mg	420 mg	104%
Phosphorus	1160.836 mg	1250 mg	93%
Potassium	4776.004 mg	4700 mg	102%
Selenium	37.492 mcg	55 mcg	68%
Sodium	2338.337 mg	2300 mg	102%
Zinc	7.064 mg	11 mg	64%

The nutrient analysis results for omega-3 and omega-6 fatty acids are shown in Table 5. The meal plan provided no long-chain omega-3 fatty acids.

**Table 5:** FOK nutrient analysis results – omega fatty acids Daily Value (DV) = Reference amounts (expressed in grams, milligrams, or micrograms) of nutrients to consume or not to exceed each day. Percent Daily Value (%DV) = The average percentage of the Daily Value for each nutrient. ^ ALA has an adequate intake (AI) range for adults of 1.1 grams for females and 1.6 grams for males. n/a = not applicable

Nutrient	Meal Plan Daily Average	Daily Value (DV)	Percent Daily Value (% DV)
Omega-3 fatty acids:			
ALA (alpha linolenic acid)^	1.383 g	n/a	n/a
DHA (docosahexaenoic acid)	0.005 g	n/a	n/a
EPA (eicosapentaenoic acid)	0 g	n/a	n/a
Omega-6 fatty acids:			
LA (linoleic acid)	9.081 g	n/a	n/a

## Discussion

The following nutrients were found to be deficient in the FOK meal plan. Over time, micronutrient deficiency can have a widespread impact on the body and overall health.

Biotin

The DV for biotin is 30 mcg per day [12]. The FOK recipes met 56% of the DV (16.762 mcg). Biotin is a water-soluble vitamin that is a cofactor to enzymes in intermediary metabolism and a regulator of gene expression. Biotin is also important for fetal development. Although biotin deficiency is rare, its signs include alopecia, scaly red rash on the face and genital area, and neurologic symptoms [13].

Most foods rich in biotin are animal-derived, such as egg yolk and liver. The richest vegan source of biotin is nutritional yeast. Biotin is also found in smaller amounts in peanuts, soybeans, sunflower seeds, mushrooms and sweet potatoes, and even lesser amounts in some other vegetables and fruits. Adding nutritional yeast and other sources of biotin into the diet, or taking supplemental biotin, could help those following a vegan diet meet their daily needs.

Calcium

The DV for calcium is 1,300 mg per day [12]. The FOK recipes met 58% of the DV (756.227 mg). Approximately 72% of calcium intake in the United States comes from milk, cheese, and other dairy products or foods with added dairy ingredients [14]. Research shows that vegans typically have lower calcium intake than vegetarians or omnivores [15]. Research also suggests that people who follow a vegan diet have lower BMD and greater fracture risk [16]. A recent meta-analysis indicated that plant-based diets are correlated with lower BMD, particularly among females when compared with an omnivore population [17].

Adequate calcium is required for proper nerve and muscle function, bone density, blood vessel contraction and dilation, blood clotting, and hormone secretion. Calcium deficiency can lead to osteoporosis, osteomalacia, or rickets [18]. 

Consuming plant sources of calcium such as foods made from soybeans (tofu, tempeh), other beans and legumes, almonds, seaweed, dark leafy greens (spinach, bok choy, mustard, collard, and turnip), blackstrap molasses, and fortified foods and beverages (plant milks, orange juice) can help those following a vegan diet meet their daily needs. Vegans who do not carefully monitor the calcium content of their diet and do not consume adequate calcium from food may need to add calcium supplementation to meet their needs.

Choline

The DV for choline is 550 mg per day [12]. The FOK recipes met 30% of the DV (162.820 mg). Choline is an essential nutrient for proper liver, muscle, and brain function, lipid metabolism, and cellular membrane composition and repair. Choline and its metabolites are involved in cell signaling, nerve transmission, and methyl donation. Although a small amount of choline is synthesized in the liver, adequate intake is required to prevent deficiency [19]. Choline deficiency results in fatty liver, liver damage, and muscle damage [20].

While choline is essential through all life stages, it is particularly important during pregnancy and lactation for fetal and infant development. As a result, the American Medical Association recommends that all prenatal vitamin supplements contain choline [21].

Animal-derived foods generally contain more choline than plant foods. Naturally rich animal sources include beef, chicken, egg yolks, fish, and pork. Rich plant sources of choline include foods made from soybeans (tofu, soynuts, and soymilk), cruciferous vegetables, cooked dried beans, and peanuts or peanut butter [20].

Although studies have not been done to determine choline intake and status of vegans and vegetarians, a recent study found that vegan and dairy-free vegetarian menus, based on the Dietary Guidelines for Americans, did not provide adequate choline [22].

Daily consumption of legumes and cruciferous vegetables can help those following a vegan diet meet the DV for choline. For vegetarians, consuming eggs and dairy can also help meet choline needs. Depending on their dietary intake, some vegans may need to take a choline supplement.

Iodine

The DV for iodine is 150 mcg [12]. The FOK recipes, which recommend the use of sea salt exclusively, met only 1% of the DV (1.4 mcg). Iodine is an essential trace mineral required to make thyroid hormones triiodothyronine (T3) and thyroxine (T4), which are involved in metabolism and protein synthesis. Iodine is also essential for fetal neurodevelopment. Potential consequences of iodine deficiency include goiter, hypothyroidism, cretinism, and impaired cognitive development [23].

The top dietary sources of iodine in the United States are dairy products and iodized salt, although seafood is also a rich source. The only plant source of naturally occurring iodine is seaweed. Himalayan salt, sea salt, and kosher salt do not contain iodine, and replacing iodized table salt with these specialty salts can potentially increase the risk of iodine deficiency.

Research suggests that vegans have an increased risk of low iodine status, deficiency, and inadequate intake compared to omnivores and even vegetarians [24]. Those following a vegan diet can increase their intake of iodine by incorporating iodized salt into their diet or adding a daily multivitamin and mineral supplement with iodine.

Iron

The FOK recipes provided 21.67 mg of iron, which exceeded the DV (18 mg) [12]. However, a recent review found that vegetarians have a high prevalence of depleted iron stores according to ferritin levels and have a higher risk of developing low iron stores, iron depletion, and iron deficiency anemia than non-vegetarians [25]. This is likely due to the lower bioavailability of non-heme iron in plant foods compared to heme iron in animal foods. To compensate, it has been suggested that vegetarians and vegans require 1.8 times more iron than those who consume meat [12]. If the DV for adult vegans and vegetarians was recalculated to meet these increased needs, the FOK recipes would only meet 67% of the “adjusted” DV.

Those following a vegan diet can meet their iron needs by consuming plant sources of iron such as leafy greens, legumes, nuts, and seeds. Pairing these iron-rich foods with vitamin C-rich foods increases non-heme iron absorption. Iron supplementation may also help vegans and vegetarians meet their daily needs.

Niacin

The DV for niacin is 16 mg [12]. The FOK recipes provided 75% of the DV (14.947 mg). Niacin (vitamin B3) is a water-soluble vitamin that is converted to its main metabolically active form, the coenzyme nicotinamide adenine dinucleotide (NAD), in the body. NAD is required by hundreds of enzymes to catalyze reactions in the body. NAD is further converted into another active form, nicotinamide adenine dinucleotide phosphate (NADP). NAD and NADP are required in many metabolic processes in cells.

Animal-based foods (poultry, beef, and fish) have about 5-10 mg of niacin per serving, primarily in the highly bioavailable forms NAD and NADP, while plant-based foods (nuts, legumes, and grains) contain about 2-5 mg of niacin per serving, mainly as nicotinic acid. The amino acid tryptophan can also be converted to NAD when present in amounts greater than required for protein synthesis. Although the main dietary sources of tryptophan are animal foods such as poultry, beef, pork, milk, and eggs, it is also found in plant foods such as soybeans (tofu and edamame), pumpkin seeds, and oatmeal [26].

Although niacin deficiency is rare in the U.S., some individuals may have marginally low niacin status. Those who do not consume adequate niacin from foods, and do not get adequate amounts of other nutrients needed to convert tryptophan to niacin (riboflavin, pyridoxine, or iron) may be at an increased risk of inadequacy [26]. Consuming niacin-rich plant foods can help those following a vegan diet meet their daily needs.

Selenium

The DV for selenium is 55 mcg [12]. The FOK recipes provided 68% of the DV (37.492 mcg). Selenium is an essential trace mineral that is critical for reproduction, thyroid hormone metabolism, DNA synthesis, and protection from oxidative damage and infection. The richest sources of selenium are Brazil nuts, seafood, and organ meat, although the main sources of selenium in the U.S. diet are breads, grains, meat, poultry, fish, and eggs. While various plant foods contain selenium, the amount depends on the amount of selenium in the soil and several other factors, including soil pH and the amount of organic matter in the soil. As a result, the amount of selenium in plant-based foods varies greatly by geographic location [27].

Research indicates that vegan diets are low in selenium [28]. Consuming just a few Brazil nuts each week, along with other plant sources of selenium, can help those following a vegan diet meet their daily needs.

Vitamin B12

The DV for vitamin B12 is 2.4 mcg [12]. The FOK recipes provided 82% of the DV (1.959 mcg). Vitamin B12 is a water-soluble vitamin that is required for central nervous system function, red blood cell formation, and DNA synthesis [29]. Vitamin B12 naturally occurs in animal foods such as meat, fish, poultry, eggs, and dairy products. Plant foods do not naturally contain vitamin B12. However, some breakfast cereals, nutritional yeast, and plant beverages such as almond milk and soymilk are fortified with vitamin B12. Many of the FOK recipes included vitamin B12-fortified plant beverages.

Studies have shown that vegan diets are low in vitamin B12 [9] and individuals consuming a plant-based diet have lower vitamin B12 status as compared to meat eaters [10]. Inadequate intake of vitamin B12 over time can result in vitamin B12 deficiency and its many adverse effects, including megaloblastic anemia, low white and red blood cells and platelets, fatigue, heart palpitations, and neurological changes [29]. To ensure adequate vitamin B12 intake and status, vegans and vegetarians need to consume vitamin B12 through fortified foods or dietary supplements.

Vitamin D

The DV for vitamin D is 20 mcg [12]. The FOK recipes provided only 5% of the DV (1.006 mcg). Vitamin D plays a critical role in calcium absorption and bone health. Research indicates that vitamin D is also involved in immune system regulation and potentially decreases the risk of a variety of health conditions. Vitamin D deficiency has been linked to various cancers (breast, colorectal, and others), heart disease and cardiovascular disease mortality, macular degeneration, falls in the elderly, depression, impaired immune function, type 2 diabetes, and other chronic diseases [30]. In addition, low 25(OH)D levels have been associated with lower pregnancy rates in women trying to conceive and a higher risk of pregnancy complications such as gestational diabetes [31, 32]. Vitamin D supplementation during pregnancy has been shown to decrease the risk of complications such as preeclampsia, low birthweight, and preterm birth [33, 34].

Few foods are naturally rich in vitamin D, and most are from animals. Fatty fish, cod liver oil, and egg yolks are good sources. Dairy milk and some other beverages are fortified with vitamin D. Mushrooms can produce vitamin D in response to sunlight or UV lamp exposure, but they make primarily vitamin D2. The more bioavailable form, vitamin D3 [35], is produced in the skin with adequate sunlight exposure. However, several factors impact how much vitamin D is made, including environmental conditions, skin pigmentation, clothing, sunscreen use, geographic location, and body weight [36].

Although the DV for vitamin D is 20 mcg (800 IU), many vitamin D experts recommend higher doses to support optimal 25(OH)D levels, especially for those at risk of vitamin D deficiency [37]. Due to the limited plant-based sources of vitamin D3, those following a vegan diet will likely need to supplement with vitamin D3 to meet their daily needs.

Vitamin E

The DV for vitamin E is 15 mg [12]. The FOK recipes provided only 7% of the DV (1.317 mg). Vitamin E is a fat-soluble vitamin that is naturally occurring in foods in eight chemical forms with different levels of biological activity. Vitamin E functions as an antioxidant and is involved in immune function, cell signaling, regulation of gene expression, and other metabolic processes [38].

Rich sources of vitamin E (alpha-tocopherol) include nuts, seeds, and vegetable oils. Green leafy vegetables and fortified cereals are also a significant source. Most vitamin E in U.S. diets is in the form of gamma-tocopherol from soybean, canola, corn, and other vegetable oils [38].

Vitamin E deficiency is rare. Because vitamin E requires fat for absorption and is found in foods rich in fat, it is not surprising that a low-fat vegan diet such as recommended by FOK is low in vitamin E. Adding more nuts, seeds, and oils to the diet could help those following a vegan diet meet their daily vitamin E needs.

Zinc

The DV for zinc is 11 mg [12]. The FOK recipes provided 64% of the DV (7.064 mg). Zinc is an essential mineral that is involved in the catalytic activity of enzymes, protein and DNA synthesis, cell division, immune function, wound healing, sense of tase and smell, and growth and development. Signs of zinc deficiency include growth retardation, impaired immune function, hair loss, diarrhea, poor wound healing, and loss of appetite [39].

A systematic review and meta-analysis of 26 studies found that zinc intakes and serum zinc concentrations were significantly lower among vegetarians compared with non-vegetarians, with greater impact on zinc intake and status of females and vegans [40]. This may be because the bioavailability of zinc is lower in plant versus animal foods such as red meat, poultry, and seafood. Although there are several plant-based sources of zinc (legumes, whole grains, nuts, and seeds), these foods contain phytates, which decrease the bioavailability of zinc [41]. Thus, vegans and vegetarians may require up to 50% more zinc than non-vegetarians [21]. Vegans and vegetarians may need to take supplemental zinc to help meet their needs.

Omega-3 Fatty Acids

The FOK recipe analysis provided 1.383 g of the essential omega-3 fatty acid alpha-linolenic acid (ALA). There is no DV for ALA; however, the Food and Nutrition Board of the National Academy of Medicine has established an adequate intake (AI) range for adults of 1.1 g for females and 1.6 g for males [42]. The FOK recipes exceeded the AI for women, but only provided 86% of the AI for men.

ALA is an essential omega-3 fatty acid and must be consumed daily as it cannot be made by the body. Vegan diets can provide significant amounts of ALA, as flaxseeds, walnuts, chia seeds, and plant oils are all rich sources. However, low-fat diets such as those recommended by FOK may limit the intake of nuts and seeds, resulting in low ALA intake.

Omega-3 fatty acids are important structural components of cell membranes in the body and serve as precursors involved in cell functioning (signaling and regulation) and provide a source of energy. Two other important omega-3 fats are the long-chain omega-3 fatty acids, eicosapentaenoic acid (EPA) and docosahexaenoic acid (DHA).

ALA can be converted to EPA and DHA, but even under the best conditions, this conversion is minimal. Research shows that about 5%-8% of ALA is converted to EPA and DHA. Excess linoleic omega-6 fatty acid intake suppresses this conversion further by competing for the elongation and desaturase enzymes [43]. As a result of limited conversion, studies have shown that vegans and vegetarians have lower plasma concentrations of EPA and DHA when compared to omnivores who eat fish [44].

A 2022 review investigated the bioavailability of plant omega-3 oils, including the conversion of ALA to EPA and DHA. This study found that high-dose flaxseed or echium seed oil supplements (rich in ALA) resulted in either no change or a reduction in the omega-3 index. However, algal oil EPA and DHA supplementation increased omega-3 index levels in all studies [45].

EPA and DHA have shown numerous health benefits, including vision and brain development, decreasing inflammatory markers, helping with athletic recovery, and lowering triglyceride levels [42,46,47]. Vegans and vegetarians could potentially benefit from an algae-based long-chain omega-3 supplement to increase their intake and blood levels of EPA and DHA. The potential adverse effects of marginal deficiency are summarized in Table 6.

**Table 6:** Potential health impact of marginal micronutrient deficiency

Nutrient	Potential Health Impact of Marginal Deficiency
Biotin	Increased birth defect risk, neurological symptoms [13]
Calcium	Decreased bone mineral density, accelerated bone loss, increased fracture and osteoporosis risk [14]
Choline	Non-alcoholic fatty liver disease, increased risk of neural tube defects, impaired neurocognitive development [20]
Iodine	Impaired fetal neurodevelopment, hypothyroidism [23]
Iron	Fatigue, anemia, pregnancy complications [25]
Vitamin B12	Increased homocysteine levels, megaloblastic anemia, fatigue, neurologic symptoms [29]
Vitamin D	Impaired immunity, adverse pregnancy outcomes, increased risk of osteoporosis, autoimmune, cardiovascular, and neurodegenerative diseases [30, 32]
Vitamin E	Impaired immunity [38]
Zinc	Impaired immunity and sense of smell, adverse pregnancy outcomes [39]
Omega Fatty Acids	Rough, scaly skin and dermatitis [46]

## Conclusions

A nutrient analysis of a one-week 2,000-calorie vegan meal plan comprised of randomly selected FOK recipes found that even a well-planned, whole-food vegan diet can be deficient in several important micronutrients. In addition, some nutrients from plant-based sources are not as bioavailable as those from animal-based sources, suggesting that an adjusted recommended intake for certain nutrients, i.e., iron, zinc, and omega-3 fatty acids, should be considered for vegetarian and vegan adults when determining their specific needs.

We acknowledge possible limitations to this study, including that the recipes are from a single source. Although FOK is a trusted source in the vegan community, other sources of vegan recipes could result in different nutrient analyses. In addition, this analysis was based on a 2,000-calorie intake. For individuals consuming greater or less than 2,000 daily calories, their nutrient intake will differ from these results.

In conclusion, although a vegan diet can provide many health benefits, even with carefully considered meal planning it is likely to be inadequate in multiple nutrients. Including fortified foods such as plant-based beverages, adding more nuts and seeds, and replacing sea salt with iodized salt could help prevent some micronutrient deficiencies in those following this vegan meal plan. In addition, nutritional supplementation with certain nutrients will help vegans meet their daily nutrient needs and potentially prevent nutrient deficiencies and resulting health consequences.
